# Phenolic Acid Profiles and Antioxidant Activity of Major Cereal Crops

**DOI:** 10.3390/antiox9060527

**Published:** 2020-06-16

**Authors:** Daniela Horvat, Gordana Šimić, Georg Drezner, Alojzije Lalić, Tatjana Ledenčan, Marijana Tucak, Hrvoje Plavšić, Luka Andrić, Zvonimir Zdunić

**Affiliations:** 1 Department—Agrochemical Laboratory, Agricultural Institute Osijek, Južno predgrađe 17, 31000 Osijek, Croatia; daniela.horvat@poljinos.hr; 2 Department of Plant Breeding and Small Cereal Crop Genetics, Agricultural Institute Osijek, Južno predgrađe 17, 31000 Osijek, Croatia; georg.drezner@poljinos.hr (G.D.); alojzije.lalic@poljinos.hr (A.L.); 3 Department of Maize Breeding and Genetics, Agricultural Institute Osijek, Južno predgrađe 17, 31000 Osijek, Croatia; tatjana.ledencan@poljinos.hr (T.L.); zvonimir.zdunic@poljinos.hr (Z.Z.); 4 Department of Forage Plant Breeding and Genetics, Agricultural Institute Osijek, Južno predgrađe 17, 31000 Osijek, Croatia; marijana.tucak@poljinos.hr; 5 Department of Seed Production and Processing, Agricultural Institute Osijek, Južno predgrađe 17, 31000 Osijek, Croatia; hrvoje.plavsic@poljinos.hr (H.P.); luka.andric@poljinos.hr (L.A.)

**Keywords:** cereals, wheat, barley, corn, phenolic acids, HPLC, antioxidant activity

## Abstract

Phenolic acids (PAs) are a dominant group of phenolic compounds in cereals, existing mostly bound to compounds of cell wall. In this study, a total of 25 cereal grain samples, including wheat, winter and spring barley, corn, and popcorn, were evaluated for bound PAs and antioxidant activity in a two-year field trial. The PA contents, determined by HPLC, were significantly affected by cereal type. The mean total PA content was highest in popcorn and corn (3298 and 2213 μg/g_dm_, respectively_)_, followed by winter and spring barley (991 and 908 μg/g_dm_, respectively) and wheat (604 μg/g_dm_). Ferulic acid was the most abundant, accounting from 62% to 83% of total PAs (in popcorn and winter and spring barley, respectively). Across cereals, p-coumaric (35–259 μg/g_dm_) and p-hidroxybenzoic (45–79 μg/g_dm_) were also dominant, while in corn and popcorn o-coumaric (71 and 89 μg/g_dm_, respectively) also occurred in higher content. The mean total phenol content ranged from 853 μg GAE/g_dm_ (wheat) to 1403 μg GAE/g_dm_ (winter barley) with DPPH scavenging activity from 14% to 67%, respectively. A significant influence of crop years on the ferulic acid and total PA content was found, while the variability of other PAs was dependent on the cereal type. The results indicated a high health benefit potential of selected cereals.

## 1. Introduction

Growing of cereals has a long tradition in the Europe. The most widely-grown cereal crop in the European Union (EU) is wheat, accounting for more than half of the total cereal production. The remaining 50% is composed of corn and barley, each representing about one third. Oats, rye, and triticale are also of significance, while other cereals are grown in smaller quantities. Cultivation of specific cereal crops varies among European countries due to its diverse climatic and pedological conditions, overall agricultural systems, wide cultural diversity, and nutritional habits. The majority of cereal harvest within the EU is used for animal feed (nearly two thirds); one third is directed at human consumption [1,2]. In recent years, many clinical trials and observational and research studies have supported the strong relationship between a diet rich in wholegrain foods and a lower incidence of chronic diseases like type 2 diabetes and cardiovascular disorders in humans. The finding that cereals contain high amounts of phenolic compounds, which are mainly phenolic acids, indicates that consumption of whole grains might contribute to the maintaining of public health. Phenolic acids found in cereals are grouped into derivatives of benzoic acid (e.g., *p*-hydroxybenzoic, protocatechuic, and vanillic acids) and cinnamic acid (e.g., *p*-coumaric, caffeic, ferulic, and sinapic acids). The majority of these exist in bound form, esterified to cell wall material in bran. Ferulic acid is the most abundant phenolic acid in cereals with 75% found in the kernel husk, 15% in the grain endosperm, and the rest in the aleurone layer [3,4]. Concerning the antioxidant activity of major cereals’ phenolic acids, the capacity of these acids to bind free radicals declines as follows: gallic, caffeic, benzoic, sinapic, syringic, ferulic, p-coumaric, vanillic, and 4-hydroxybenzoic [4].

Phenolic acid (PA) profiles and the content of whole grain or grain fractions have mainly been studied in wheat and to a limited extent in barley and corn, so comparison of phenolic acid profiles of major cereal crops under similar experimental conditions is needed [5]. Despite relatively high genetic impact, some authors have shown that environment factors, including weather conditions during the growing period (water and temperature stress), have a strong influence on the selected health-beneficial components of cereals and their antioxidant properties [6,7,8]. Cereal varieties developed in recent years have been more oriented to crop yields and height, timing of crop maturation, protein content, and disease and pest resistance. In order to obtain products of high health-promoting value it is essential to modify the contents of bioactive compounds at every stage of plant production, starting from growing crops, through all the technological processes to the final product [4]. In Croatian cereal production, corn takes 51.5%, wheat 29.4%, and barley 10.3% and other cereals are represented by 8.8%. The Republic of Croatia is self-sufficient in the production of cereals, except in years with extremely unfavourable climatic conditions [9]. Cereal research has been traditionally focused on agronomic and industrial quality, however, recently, more attention at the Agricultural Institute Osijek has been paid to phytochemicals which are positively associated with human health.

Many articles deal with characterization of grain phenolic compounds and antioxidant capacity of specific cereal genera [4,6,7,10]. Only a few studies are related to the analysis of complex bound PAs including both small grain cereals and corn [3,5]. Therefore, the aim of this study was to present a comprehensive approach to profiling of phenolic acids in major cereal crops created at the Agricultural Institute Osijek and grown over two crop years. Characterization of the most common phenolic compounds in cereals opens new opportunities for breeding and commercial production of value-added cultivars rich in beneficial components and their utilization in functional foods.

## 2. Materials and Methods

### 2.1. Chemicals

Chemical materials used in this study, including phenolic acid standards, were of analytical or HPLC-grade and were sourced from KEFO (Sisak, Croatia). Water for analyses was purified by the Milli-Q system (Millipore Corp., Milford, MA, USA).

### 2.2. Plant Materials

A total of 25 samples of major cereal crops, including wheat (*Triticum aestivum* L.), winter and spring barley (*Hordeum vulgare* L.), and corn (*Zea mays* L.), were evaluated for phenolic acids over two years (2018 and 2019). Small grain cereals comprised three sets of five cultivars: bread wheat (Kraljica, Olimpija, Srpanjka, Katarina, and Renata), winter (Maxim, Bravo, Maestro, Barun, and OS Lukas), and spring barley (Igor, Fran, Matej, Patrik, and Pivarac). Two types of corn were chosen, five hybrids of yellow dent (Kulak, OS 398, OS 378, Tomasov, and Velimir) and four hybrids and one breeding line of popcorn (OS POP 555, OS POP 566, Bulut, OS 12XDH4-2, and OS 504pc).

Grain samples were collected after harvesting of crops grown in 2018 and 2019 in the field trials at the Agricultural Institute Osijek (45°33′20″ N 18°41′40″ E, 94 m altitude, average annual temperature 11.0 °C, and average precipitation 655 mm). Standard agronomic practices were applied for each cereal crop over two consecutive years. Wheat and winter barley were sown in October of the year prior to harvest, spring barley in February and corn in April. In 2018, average annual temperature and precipitation were 13.1 °C and 650 mm, respectively, while in 2019, their values were 12.9 °C and 759 mm, respectively (Table 1).

The whole-grain cereal flours were prepared by grinding in a hammer type cyclone mill (Laboratory Mill 3100; Perten Instruments AB, Sweden) equipped with 0.75 mm sieve.

### 2.3. Extraction of Phenolic Acids for HPLC Analysis

Extraction of bound phenolic acids was done according to Zavala-Lopez and Garcia-Lara [10]. Necessary modifications were applied to adjust procedure to the laboratory conditions. Ester-linked phenolic acids were extracted from 0.25 g of cereal whole-grain pellet residue after discharging of free-form phenolic acids. Alkaline hydrolysis was performed with 2.5 mL of 2 M NaOH at 90 °C for 2 h. Following hydrolysis, extract was acidified with 2.5 mL of 2 M HCl at pH 2 and lipids were removed with n-hexane. Ethyl acetate was applied three times for recovering phenolic acids, then evaporated to dryness and stored at −20 °C in methanol suspension. All extraction procedures were done in duplicates.

### 2.4. HPLC Analysis of Phenolic Acids

HPLC analysis of phenolic acids was done by using a Series 200 HPLC system (Perkin Elmer, USA) equipped with a Kinetex Core-Shell RP-C18 column and detected by diode array detector (DAD). The mobile phase consisted of two solvents, purified water and acetonitrile acidified with 1% trifluoroacetic acid (*v/v*). Gradient elution from 5% to 40% acetonitrile solvent in 40 min was applied. The column was equilibrated between two runs for a period of 5 min with 90% isocratic acetonitrile. According to comparison to retention times of external standards at 275 nm the following phenolic acids were identified: p-hydroxybenzoic, vanillic, caffeic, syringic, p-coumaric, ferulic, and o-coumaric acid. Quantification of individual phenolic acids was done using a 5-point calibration curve (*R*^2^ ≥ 0.999) following retention times and DAD absorption spectra of external standards. Composition analysis of each extract was carried out in duplicate.

### 2.5. Extraction Procedure for Total Phenolic Content and Antioxidant Activity Assays

Total phenolic content (TPC) from cereal grains was extracted according to the modified method of Singleton and Rossi [11]. Flour (1 g of cereal whole-grain meal) was mixed with 3 mL of 0.1% HCL in methanol. The mixture was homogenized for 2 min and sonicated (Sonorex Digitec, Bandelin, Germany) for 60 min. After centrifugation (Universal 320R; Hettich, Germany) at 9000 rpm for 5 min at 4 °C, the supernatant was removed, and extraction procedure of the residue was repeated with 3 mL of acidified methanol and sonicated 30 min. The supernatants were pooled and stored in the dark at –20 °C until analysis.

### 2.6. Total Phenolic Content Assay

The total phenolic content (TPC) in cereal extracts was determined with Folin−Ciocalteu reagent according to Singleton and Rossi [11] with some modifications. Briefly, 0.1 mL of extract reacted with 0.1 mL of Folin-Ciocalteu phenol reagent (1:1) and 1.5 mL of distilled water. It was homogenized and after 5 min 0.3 mL of 20% Na_2_CO_3_ solution was added. The mixture was thoroughly shaken and incubated for 60 min at room temperature in a dark place. Total phenolics were quantitated by spectrophotometric method at 765 nm. The analyses were performed in triplicate. The results were quantified using external calibration and expressed as μg of gallic acid equivalent (GAE) per g of dry matter.

### 2.7. Antioxidant Activity Assay

Antioxidant activity was determined using free radical 2,2-diphenyl-1-picrylhydrazyl (DPPH) radical scavenging assay. Antioxidant activity was measured using a modified version of the method of Brand-Williams et al. [12]. For each measurement, 0.2 mL was taken from the sample extract and a mixture was formed by adding 1 mL of a 0.5 mmol/L methanol solution of DPPH and 2 mL of methanol. After incubation for 30 min in a dark place, the absorbance was determined at 517 nm. The procedure was repeated three times. The percentage of inhibition of free radical DPPH in percent (%) was calculated against blank:

% Inhibition = (1 − (A sample_t = 30_/A blank_t = 0_)) × 100

### 2.8. Statistical Analysis

The Paleontological Statistics Software Package was applied to perform one-way ANOVA statistical analysis of experimental data. Fisher’s least significant difference (LSD) test (*p* < 0.05) was used to create confidence intervals for all pairwise differences between means.

## 3. Results and Discussion

### 3.1. Phenolic Acids

#### 3.1.1. Cereals

Phenolic acids (PAs) are the most prominent and well-characterized phenolic compounds in cereal grains [3]. Statistically-significant differences in PA content occurred across cereal crops analysed in this study (Table 2). On average, the highest total PA content was determined in popcorn (3298 µg/g_dm_) and corn (2213 µg/g_dm_), followed by winter and spring barley (991 and 908 µg/g_dm_, respectively) and wheat (604 µg/g_dm_).

Consequently, ferulic acid (FER) was found to be significantly higher in popcorn and corn extract (2741 and 1748 µg/g_dm_, respectively) compared to wheat (448 μg/g_dm_) and barley (610 and 568 μg/g_dm_, respectively). FER accounted for approximately 62% of total bound PAs in winter and spring barley, 74% in wheat, and the highest, 78% and 83% in corn and popcorn. The major PAs were p-COU (35–259 μg/g_dm_) and p-hydroxybenzoic acid (p-HB) (45–79 μg/g_dm_), while a larger amount of o-coumaric acid (o-COU) was found in corn and popcorn (71 and 89 μg/g_dm_, respectively) (Table 2). It has been previously reported that the content of bound FER and total PAs was highest for corn in comparison to other cereals [13].

#### 3.1.2. Wheat

Wheat (*Triticum aestivum* L.) is one of the major food grains consumed by people. Although wheat is used mainly as a source of energy, whole wheat grains are an excellent source of dietary fibre, vitamins, minerals and other bioactive phytochemicals such as antioxidant compounds [3]. Five wheat cultivars, which are among the most cultivated in Croatia because of their medium to high yield combined with good quality of the flour, were used in this study (Table 3). There were significant differences (*p* < 0.05) in PA content among wheat cultivars. FER, in the range from 424 (Kraljica) to 482 µg/g_dm_ (Katarina), was the most abundant PA in wheat grains (Table 3), which is in accordance with other authors [3,4]. Mpofu et al. [14] reported FER content across cultivars from 371 to 441 μg/g. Similar FER concentrations were found in studies of Hung et al. [15] and Zhang et al. [16]. In agreement with others [3,4], p-HB, p-COU, caffeic acid (CAF), syringic acid (SYR), o-COU, and vanillic acid (VAN) were also found in smaller concentrations (Table 3).

The total content of bound PAs varied between 556 and 654 µg/g_dm_ what corresponds with Yilmaz et al. [17] and Leváková and Lacko-Bartošová [18], who found in selected wheat species, total PAs in the range 510–831 µg/g_dm_ and 516 and 831 μg/g_dm_, respectively.

The PA content of wheat is significantly influenced by weather conditions during the growing seasons (Table 3). In contrast to FER, total PA content was higher (*p* < 0.05) in 2018, compared to 2019, because of the opposite reaction of minor PAs. Many antioxidants produced by plants respond to abiotic stress, like water and heat stress [19]. Zrckova et al. [20] recorded significant changes between two crop years of wheat and concluded that the higher total PA contents were related to lower rainfall and higher temperatures during the ripening stages of cereals.

#### 3.1.3. Barley

Barley (*Hordeum vulgare* L.) is the fourth-most important cereal in terms of world production and 80–90% of barley production is used for malting and animal feedstock [3]. The abundant content of phenolic compounds in barley reveals that it may serve as an excellent dietary source of natural antioxidants with antiradical and antiproliferative potentials for disease prevention and health promotion [21]. Significant differences (*p* < 0.05) between winter and spring barley cultivars were found for p-HB, VAN, CAF, p-COU, and FER (Table 2). FER was found to be the most abundant PA in both barley types, ranging from 549 (OS Lukas) to 667 µg/g_dm_ (Barun). p-COU and p-HB acid were the second- and third-most abundant PAs according to existing concentrations in barley samples, from 122 (Fran) to 271 µg/g_dm_ (OS Lukas) and from 46 (OS Lukas) to 80 µg/g_dm_ (Patrik), respectively (Table 3). According to data published by Arigò et al. [22] and Martínez et al. [23], these PAs were the major phenolic acids in hull-less and hulled barley. Further, regarding ferulic acid content, we found our results to be similar to those of Ndolo and Beta [5] who found FER in hulled barley at a concentration of 731 μg/g. Gamel and Abdel-Aal [24] noted FER content in the range of 124–466 μg/g in selected hulled barley cultivars, similarly to others [6,25], while Gałązka et al. [26] reported FER content in winter barley in a much higher range (1158–1435 μg/g).

The amount of p-COU, as the second-most major PA in barley samples, was at a similar level to that in a study by Galazka et al. [26] (98.6–201.41 μg/g) and much higher than reported by Legzdina et al. [6] 3.83–70.64 μg/g (Table 4). A significant variability of total PA was observed among the whole-grain barley samples with values from 828 to 1056 µg/gdm (Table 4).

Significant differences between two crop years were found for p-COU, FER, o-COU, and total PAs in both barley types and the concentration values were higher (*p* < 0.05) in 2019 compared to 2018 (Table 4). Limited research results are published on the effect of weather conditions on barley phenolics. Legzdina et al. [6] found that weather conditions during the spring barley vegetation period were highly significant and that a cooler crop year promoted higher concentrations of ferulic, *p*-coumaric, 4-hydroxybenzoic, chlorogenic, and gallic acids. In our study, in May 2019 (earlier grain-filling stage), when, according to Ma et al. [7], intensive synthesis of PAs occurs, 5.5 times more rain fell and the temperature was 6.6 °C lower compared to May 2018 (results were not shown). It could be possible to assume that in 2019, when the total PA was significantly higher, evaluated barley varieties were exposed to higher water stress.

#### 3.1.4. Corn and Popcorn

Corn (*Zea mays* L.) is an edible cereal plant widely grown and consumed in many different food products, alone or as a part of various recipes [27]. Among domesticated corn, popcorn makes a promising crop rich in phytochemicals since it is a popular whole-grain snack food. Significant differences (*p* < 0.05) between corn and popcorn samples were found for most PAs (Table 2). FER was the major PA in both corn types with a range from 1603 µg/g_dm_ (Tomasov) to 3059 µg/g_dm_ (OS 12XDH4-2), followed by p-COU and p-HB in the range of 188 µg/g_dm_ (Kulak) to 329 µg/g_dm_ (OS 378) and from 29 µg/g_dm_ (Kulak) to 105 µg/g_dm_ (OS 378), respectively (Table 5). Our results of FER content in corn are similar to those of Čukelj et al. [28] and Rodriguez-Salinas et al. [29]. Trehan et al. [30] showed the presence of FER in different corn accessions in a much wider range (1860–6275 μg/g).

Cuevas-Montilla et al. [31] in different Bolivian purple corn found FER in the range from 1329–2984 μg/g. There is a lack of available data about PA profiles in popcorn. Kumar and Pruthi [32] noted that popcorn contained FER in the amount of 3130 μg/g, what is similar to our results.

The amounts of PAs were statistically different in the two crop years. Total PAs in corn and popcorn were higher in 2019 (2296 and 3445 μg/g_dm_, respectively) in comparison to 2018 (2130 and 3152 μg/g_dm_, respectively) (Table 5). As we mentioned above, in 2019, cereals were exposed to higher water stress. There is a lack of literature data on environmental (climate conditions) impact on PA synthesis in corn, so in this research area further studies are required.

### 3.2. Antioxidant Activity (AOA)

#### 3.2.1. Total Phenolic Content (TPC)

**Figure 1:**
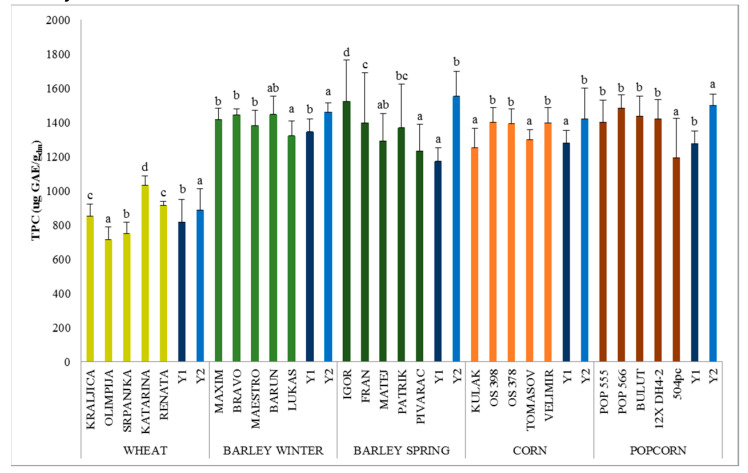
Total phenolic content (μg GAE/g_dm_) in cereal grains. Mean ± SD of 2 years and triplicate extractions (*n* = 6). Bars with different superscript (a–d) letters are significantly different (*p* ≤ 0.05).

The Folin-Ciocalteu (F-C) assay is widely used to evaluate the in vitro TPC of cereal extracts. Free phenolic compounds are the major contributors to the total AOA in methanol and methanol/HCl extracts [22], which means that only soluble polyphenols extracts were used for the evaluation of antioxidant activity in our study, while insoluble bound phenolic compounds that are esterified to macromolecules were not taken into consideration. Among analysed cereals, the highest variability (*p* < 0.05) was found within wheat varieties/samples and the lowest within corn and popcorn (Figure 1). On average, the highest TPC was found in winter barley (1322–1448 μg GAE/g_dm_) and popcorn (1195–1486 μg GAE/g_dm_), and the lowest in wheat (713–1032 μg GAE/g_dm_) (Figure 1). To facilitate the comparison of cereals, the same extraction procedure of TPC was used in our study. Solvent concentrations, solvent to sample ratio, pH, and temperature can significantly affect the total polyphenols recovery [10].

Paznocht et al. [8] published the TPC of coloured wheat in the range 599–798 μg GAE/g. In the study of Legzdina et al. [6], TPC in hulless and hulled spring barley ranged between 817 and 1401 μg GAE/g, while Šimić et al. [33] reported TPC content in hulled barley in the range from 1270–1670 μg GAE/g_dm_. Trehan et al. [30] and Yilmaz [34] reported that TPC in yellow and white corn ranged between 903–1640 μg GAE/g and 816–948 μg GAE/g_dm_, respectively. Niroula et al. [35] found in corn the lowest TPC content (985 μg/g_dm_) in comparison to wheat (1091 μg/g_dm_) and barley (1274 μg/g_dm_). Coco and Vinson [36] in a recent study reported that on average nine commercial popcorn samples contained 2660 μg/g of TPC after in vitro digestion by Folin-Ciocalteu test, and also noted that TPC levels in popcorn samples were similar to those in other studied corn. We also had similar results.

#### 3.2.2. DPPH Radical Scavenging Activity

**Figure 2:**
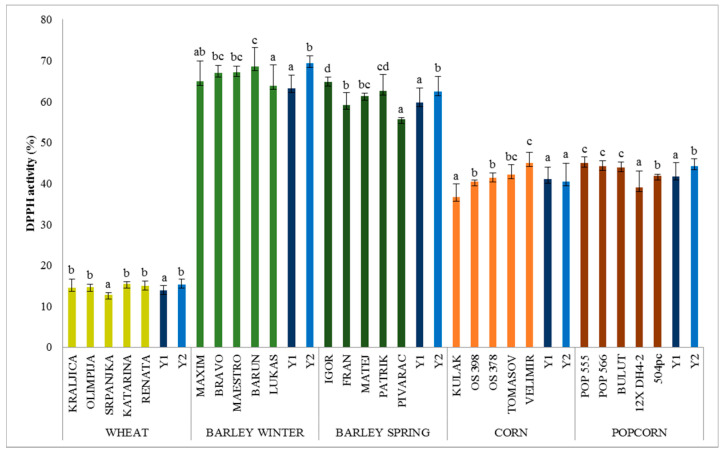
DPPH radical scavenging activity (%) in cereal grains. Mean ± SD of 2 years and triplicate extraction (*n* = 6). Bars with different superscript letters (a–d) are significantly different (*p* < 0.05).

Antioxidant activity of the methanol extract of cereal grains is widely evaluated using a free DPPH radical scavenging assay. The DPPH radical scavenging assay is easy to use because the DPPH radical is stable allowing antioxidants to quench its spare electron. Large differences were noticed between the antioxidant activities among cereals (Figure 2). The highest DPPH scavenging activity was found in winter and spring barley (64–69% and 56–65%, respectively), followed by popcorn (39–45%), corn (37–45%), and wheat (13–15%) (Figure 2). There are a number of publications that have reported on the antioxidant activity of cereal extracts, but it is difficult to compare results because of different extraction protocols and assays tests used. Similarly to our results, Mpofu et al. [14] and Sandhu et al. [37] reported DPPH values of wheat in the range 13–14% and 13–22%, respectively. Šimić et al. [33] in hulled barley noted a DPPH scavenging activity in the similar range (58–66%). A higher DPPH radical scavenging activity found in barley when compared (*p* < 0.05) to wheat is similar to findings of Žilić et al. [38]. Niroula et al. [35] reported that corn seeds had similar TPC to wheat and barley.

A significant difference between two crop years was found for TPC and DPPH scavenging activity in all cereal types and, on average, their values were higher (*p* < 0.05) in 2019 compared to 2018 (Figure 1 and Figure 2). Similarly, Holtekjølen et al. [39], Abdel-Aal and Choo [40], and Zrckova et al. [20] registered significant differences in TPC and DPPH of cereal grains depending on the varieties and yearly weather conditions.

#### 3.2.3. Correlation Between TPC and DPPH Radical Scavenging Activity

A significant positive correlation (*p* < 0.05) was found between TPC and DPPH radical scavenging in wheat (*r* = 0.598), winter and spring barley (*r* = 0.836 and 0.735, respectively), and popcorn (*r* = 0.471) (Table 6), which is in agreement with the results already published [14,34,36]. These data indicate that TPC significantly contributes to the DPPH radical scavenging activity.

There was no significant relationship between TPC and DPPH radical scavenging in corn samples (*r* = 0.202), what is in accordance with Fardet et al. [41]. A lack of correlation has been reported previously between the F-C assay and DPPH activity in cereals [39,40]. For corn samples other phytochemicals, rather than TPC, may perform a major role in radical scavenging activities [10].

## 4. Conclusions

The obtained results indicate a high antioxidant potential and health benefits of selected cereal grains. The insight into varietal and environmental effects on the phenolic acid profiles and antioxidant activity of cereal grains could be valuable information for improving breeding efforts to produce cereal grains rich in health-promoting phenolic compounds. This is of interest not only to Croatian cereal breeders, but also to the wider scientific and expert community, as well as to consumers interested in improving their nutritional habits and preferences for specific grain crops.

## Figures and Tables

**Table 1:** Monthly climate data related to the two crop years.

Month	I	II	III	IV	V	VI	VII	VIII	IX	X	XI	XII	
Precipitation (mm)													Total
2018	62	70	83	21	27	127	132	36	27	12	25	27	650
2019	42	27	8	69	151	113	57	82	75	32	57	45	759
LTP_71-00_	42	35	41	51	59	82	66	62	51	56	62	48	655
Temperature (°C)													Mean
2018	4.2	0.9	4.6	17.0	20.6	21.7	22.5	24.3	18.3	14.4	7.6	1.5	13.1
2019	0.5	4.2	9.1	12.8	14.0	23.1	22.6	23.4	17.5	13.0	10.1	4.0	12.9
LTP_71-00_	−0.2	1.8	6.4	11.2	16.7	19.6	21.3	20.8	16.5	11.1	5.1	1.3	11.0

LTP_71-00_ = Long Term Period from 1971 to 2000.

**Table 2:** Content ^1^ (µg/g_dm_) of bound phenolic acids in cereal grains.

Cereals	p-HB ^2^	VAN	CAF	SYR	p-COU	FER	o-COU	TOTAL
Wheat	45 ^a^ ± 16	13 ^a^ ± 2	27 ^a^ ± 8	20 ^a^ ± 5	35 ^a^ ± 3	448 ^a^ ± 23	17 ^a^ ± 5	604 ^a^ ± 38
Winter Barley	65 ^b^ ± 14	26 ^c^ ± 4	29 ^a,b^ ± 9	26 ^b^ ± 6	205 ^c^ ± 54	610 ^b^ ± 92	31 ^b^ ± 7	991 ^b^ ± 113
Spring Barley	68 ^b,c^ ± 32	22 ^b^ ± 3	33 ^b,c^ ± 6	30 ^b^ ± 5	161 ^b^ ± 32	568 ^a,b^ ± 78	26 ^b^ ± 7	908 ^b^ ± 68
Corn	68 ^b,c^ ± 31	24 ^c^ ± 4	34 ^c^ ± 7	29 ^b^ ± 12	238 ^d^ ± 54	1748 ^c^ ± 164	71 ^c^ ± 16	2213 ^c^ ± 239
Popcorn	79 ^c^ ± 20	32 ^d^ ± 3	29 ^a,b^ ± 9	40 ^c^ ± 10	259 ^d^ ± 41	2741 ^d^ ± 436	89 ^d^ ± 18	3298 ^d^ ± 467

^1^ Mean ± SD of five genotypes, 2 years, and duplicate extraction/HPLC analysis (*n* = 20). ^2^ p-HB = p-hydroxybenzoic acid, VAN = vanillic acid, CAF = caffeic acid, SYR = syringic acid, p-COU = p-coumaric acid, FER = ferulic acid, o-COU = o-coumaric acid, and TOTAL = sum of all phenolic acids. ^a–d^ in the same column correspond to statistically-different values (Fisher’s post hoc test *p* < 0.05).

**Table 3:** Content ^1^ (µg/g_dm_) of bound phenolic acids in wheat grains.

Varietes	p-HB ^2^	VAN	CAF	SYR	p-COU	FER	o-COU	Total
Kraljica	34 ^a^ ± 10	13 ^b^ ± 0	25 ^b^ ± 9	14 ^a^ ± 2	32 ^a^ ± 3	424 ^a^ ± 10	14 ^a^ ± 4	556 ^a^ ± 3
Olimpija	47 ^b^ ± 20	13 ^b^ ± 1	30 ^d,e^ ± 4	22 ^c,d^ ± 3	38 ^b^ ± 2	460 ^c^ ± 7	17 ^b^ ± 5	626 ^c^ ± 22
Srpanjka	49 ^b^ ± 10	11 ^a^ ± 1	26 ^c,d^ ± 9	19 ^b^ ± 4	32 ^a^ ± 1	430 ^a^ ± 12	14 ^a^ ± 3	580 ^b^ ± 6
Katarina	45 ^b^ ± 17	15 ^c^ ± 2	31 ^e^ ± 6	21 ^b,c^ ± 7	39 ^b^ ± 2	482 ^d^ ± 2	22 ^c^ ± 7	654 ^d^ ± 29
Renata	50 ^b^ ± 23	13 ^b^ ± 1	22 ^a^ ± 12	24 ^e^ ± 4	35 ^a^ ± 1	444 ^b^ ± 7	17 ^b^ ± 3	605 ^b^ ± 11
Y1 ^3^	59 ^b^ ± 10	13 ^b^ ± 2	20 ^a^ ± 6	23 ^b^ ± 4	36 ^b^ ± 4	443 ^a^ ± 26	20 ^b^ ± 4	614 ^b^ ± 47
Y2	31 ^a^ ± 5	12 ^a^ ± 2	33 ^b^ ± 1	17 ^a^ ± 3	34 ^a^ ± 3	453 ^b^ ± 19	13 ^a^ ± 2	594 ^a^ ± 26

^1^ Mean ± SD of 2 years and duplicate extraction/HPLC analysis (*n* = 8). ^2^ p-HB = p-hydroxybenzoic acid, VAN = vanillic acid, CAF = caffeic acid, SYR = syringic acid, p-COU = p-coumaric acid, FER = ferulic acid, o-COU = o-coumaric acid, and Total = sum of all phenolic acids. ^3^ Mean ± SD of each phenolic acid in year 2018 (*n* = 20) and year 2019 (*n* = 20). ^a–e^ in the same column correspond to statistically-different values (Fisher’s post hoc test *p* < 0.05).

**Table 4:** Content ^1^ (μg/g_dm_) of bound phenolic acids in winter and spring barley grains.

Varietes	p-HB ^2^	VAN	CAF	SYR	p-COU	FER	o-COU	Total
	Winter Barley							
Maxim	62 ^b^ ± 11	29 ^ns^ ± 1	21 ^a^ ± 1	26 ^b^ ± 1	225 ^b^ ± 17	565 ^a^ ± 64	26 ^a^ ± 1	954 ^a^ ± 91
Bravo	74 ^c,d^ ± 12	24 ± 2	32 ^c,e^ ± 4	34 ^c^ ± 3	203 ^b^ ± 19	659 ^c^ ± 81	30 ^b^ ± 6	1056 ^b^ ± 76
Maestro	65 ^c,d^ ± 9	24 ± 1	41 ^d^ ± 4	19 ^a^ ± 4	160 ^a^ ± 20	609 ^b^ ± 98	31 ^b^ ± 6	948 ^a^ ± 137
Barun	77 ^d^ ± 11	25 ± 0	27 ^b,c^ ± 1	30 ^b^ ± 3	168 ^a^ ± 24	667 ^c^ ± 73	40 ^c^ ± 1	1032 ^b^ ± 110
OS Lukas	46 ^a^ ± 3	28 ± 7	25 ^b,c^ ± 1	20 ^a^ ± 1	271 ^c^ ± 75	549 ^a^ ± 91	26 ^a^ ± 6	965 ^a^ ± 149
Y1 ^3^	62 ^ns^ ± 14	27 ^ns^ ± 5	31 ^ns^ ± 6	25 ^ns^ ± 4	179 ^a^ ± 30	536 ^a^ ± 54	34 ^b^ ± 5	894 ^a^ ± 65
Y2	68 ± 15	25 ± 3	26 ± 8	26 ± 8	232 ^b^ ± 59	683 ^b^ ± 52	27 ^a^ ± 6	1088 ^b^ ± 37
	**Spring Barley**							
Igor	56 ^ns^ ± 15	22 ^a,b^ ± 1	29 ^ns^ ± 6	27 ^ns^ ± 1	156 ^b^ ± 18	651 ^e^ ± 72	26 ^c^ ± 4	966 ^c^ ± 78
Fran	69 ± 31	21 ^a^ ± 4	29 ± 3	32 ± 1	122 ^b^ ± 18	529 ^b^ ± 75	26 ^b^ ± 8	828 ^a^ ± 63
Matej	65 ± 22	25 ^b^ ± 3	36 ± 7	29 ± 3	159 ^b^ ± 15	567 ^c^ ± 53	30 ^d^ ± 9	911 ^a,b^ ± 29
Patrik	80 ± 54	25 ^b^ ± 1	37 ± 4	31 ± 10	159 ^b^ ± 5	592 ^d^ ± 43	23 ^a^ ± 6	946 ^c^ ± 30
Pivarac	68 ± 36	19 ^a^ ± 1	36 ± 1	33 ± 1	207 ^c^ ± 24	500 ^a^ ± 68	24 ^b^ ± 8	887 ^a,b^ ± 48
Y1 ^3^	95 ^b^ ± 20	22 ^ns^ ± 4	34 ^ns^ ± 8	32 ^ns^ ± 5	147 ^a^ ± 27	514 ^a^ ± 59	32 ^b^ ± 4	876 ^a^ ± 69
Y2	40 ^a^ ± 5	23 ± 3	33 ± 4	28 ± 4	174 ^b^ ± 31	621 ^b^ ± 54	20 ^a^ ± 2	939 ^b^ ± 54

^1^ Mean ± SD of 2 years and duplicate extraction/HPLC analysis (n = 8). ^2^ p-HB = p-hydroxybenzoic acid, VAN = vanillic acid, CAF = caffeic acid, SYR = syringic acid, p-COU = p-coumaric acid, FER = ferulic acid, o-COU = o-coumaric acid, and Total = sum of all phenolic acids. ^3^ Mean ± SD of each phenolic acid in year 2018 (n = 20) and year 2019 (n = 20). Values with different superscript letters (a-d) in a column are significantly different (p ≤ 0.05), ns = non-significant.

**Table 5:** Content ^1^ (μg/g_dm_) of bound phenolic acids in corn and popcorn grains.

Varietes	p-HB 2	VAN	CAF	SYR	p-COU	FER	o-COU	Total
				Corn				
Kulak	29 ^a^ ± 22	20 ^a^ ± 1	33 ^a^ ± 4	18 ^a^ ± 9	188 ^a^ ± 15	1635 ^a^ ± 109	64 ^a^ ± 17	1897 ^a^ ± 104
OS 398	58 ^a,b^ ± 16	26 ^c^ ± 3	28 ^a^ ± 10	35 ^c^ ± 15	238 ^b^ ± 2	1808 ^b c^ ± 52	72 ^b^ ± 18	2266 ^b^ ± 75
OS 378	105 ^d^ ± 34	30 ^d^ ± 1	43 ^b^ ± 4	38 ^c^ ± 4	329 ^c^ ± 36	1914 ^c^ ± 32	81 ^c^ ± 18	2540 ^c^ ± 56
Tomasov	81 ^c,d^ ± 3	23 ^b^ ± 1	32 ^a^ ± 6	28 ^b^ ± 11	207 ^a,b^ ± 7	1603 ^a^ ± 88	66 ^a^ ± 14	2040 ^a^ ± 76
Velimir	67 ^b^ ± 7	22 ^a,b^ ± 2	35 ^a,b^ ± 1	26 ^b^ ± 13	230 ^b^ ± 35	1781 ^b^ ± 244	72 ^b^ ± 19	2232 ^b^ ± 283
Y1 ^3^	65 ^ns^ ± 43	24 ^ns^ ± 4	34 ^ns^ ± 9	20 ^a^ ± 9	243 ^b^ ± 64	1658 ^a^ ± 151	86 ^b^ ± 7	2130 ^a^ ± 264
Y2	70 ± 13	24 ± 4	34 ± 5	38 ^b^ ± 8	234 ^a^ ± 46	1838 ^b^ ± 127	56 ^a^ ± 6	2296 ^b^ ± 187
				**Popcorn**				
OS POP 555	51 ^a^ ± 18	33 ^ns^ ± 5	24 ^a^ ± 6	31 ^ns^ ± 3	233 ^a,b^ ± 28	2758 ^b^ ± 185	77 ^a^ ± 13	3286 ^b^ ± 336
OS POP 566	86 ^b^ ± 26	34 ± 1	24 ^a^ ± 8	41 ± 2	245 ^a,b^ ± 35	2993 ^b^ ± 295	87 ^a,b^ ± 6	3582 ^b^ ± 214
Bulut	79 ^b^ ± 5	31 ± 2	23 ^a^ ± 5	41 ± 9	228 ^a^ ± 15	2771 ^b^ ± 285	103 ^b^ ± 20	3275 ^b^ ± 268
OS 12XDH4-2	90 ^b^ ± 9	29 ± 1	39 ^b^ ± 5	47 ± 19	318 ^c^ ± 34	3059 ^b^ ± 488	104 ^b^ ± 21	3684 ^b^ ± 522
OS 504pc	89 ^b^ ± 13	31 ± 2	35 ^b^ ± 5	38 ± 4	269 ^b^ ± 4	2126 ^a^ ± 193	75 ^a^ ± 6	2663 ^a^ ± 182
Y1 ^3^	86 ^b^ ± 10	33 ^b^ ± 3	28 ^ns^ ± 12	35 ^ns^ ± 3	254 ^a^ ± 27	2555 ^a^ ± 343	97 ^b^ ± 22	3152 ^a^ ± 389
Y2	71 ^a^ ± 5	30 ^a^ ± 2	31 ± 3	45 ± 12	263 ^b^ ± 52	2927 ^b^ ± 454	81 ^a^ ± 9	3445 ^b^ ± 511

^1^ Mean ± SD of 2 years and duplicate extraction/HPLC analysis (*n* = 8). ^2^ p-HB = p-hydroxybenzoic acid, VAN = vanillic acid, CAF = caffeic acid, SYR = syringic acid, p-COU = p-coumaric acid, FER = ferulic acid, o-COU = o-coumaric acid, and Total = sum of all phenolic acids. ^3^ Mean ± SD of each phenolic acid in year 2018 (*n* = 20) and year 2019 (*n* = 20). Values with different superscript letters (a-d) in a column are significantly different (*p* ≤ 0.05), ns = non-significant.

**Table 6:** Pearson’s correlation coefficients (*r*) between total phenolic content (TPC) and DPPH scavenging activity.

Cereals	r
Wheat ^1^	0.598 *
Winter Barley	0.836 *
Spring Barley	0.735 *
Corn	0.202 ^ns^
Popcorn	0.471 *
Cereals^2^	0.750 *

^1^ Overall five varieties, 2 years, and triplicate extractions (*n* = 30). ^2^ Overall of 25 cereal varieties, 2 years and triplicate extraction (*n* = 150). * significant at *p* < 0.05, ns = non-significant.
